# JMATING: a software for the analysis of sexual selection and sexual isolation effects from mating frequency data

**DOI:** 10.1186/1471-2148-6-40

**Published:** 2006-05-09

**Authors:** Antonio Carvajal-Rodriguez, Emilio Rolan-Alvarez

**Affiliations:** 1 Department of Microbiology and Molecular Biology, Brigham Young University, 84602 Provo, Utah, USA; 2 Departamento de Bioquímica, Genética e Inmunología, Facultad de Biología, Universidad de Vigo, Campus Universitario de Vigo, 36310 Vigo, Spain

## Abstract

**Background:**

Many different sexual isolation and sexual selection statistics have been proposed in the past. However, there is no available software that implements all these statistical estimators and their corresponding tests for the study of mating behaviour.

**Results:**

JMATING is an easy-to-use program developed in Java for the analysis of mating frequency data to study sexual selection and sexual isolation effects from laboratory experiments as well as descriptive studies accomplished in the wild. The software allows the re-organization of the data previous to the analysis, the estimation of the most important estimators, and a battery of complementary statistical tests.

**Conclusion:**

JMATING is the first complete and versatile software for the analyses of mating frequency data. It is available at  and requires the Java runtime environment.

## Background

Mating behaviour is likely to be one of the most important biological processes contributing to speciation in animals [1], sexual isolation being the most obvious evolutionary strategy to impede the production of unfit hybrids when two species meet in the wild [2,3]. Studies on sexual isolation have been typically accomplished in laboratory conditions using one of four possible experimental designs: no choice, male choice, female choice and multiple choice [reviewed in [4-6]]. For simplicity we will refer always to the multiple choice design, where males and females of two or more qualitative mating types are placed in a mating chamber and mating pairs are identified. The former designs can be studied using the same estimators and methods when they use a similar number of mating attempts for every combination of mating pairs [6]. In a multiple choice experiment the mating behaviour can be disentangled into sexual isolation and selection effects [7]. This statistical partitioning has an evolutionary justification: sexual selection can change gene frequencies in populations, while sexual isolation might be directly involved in speciation [8]. In addition to these classical laboratory experiments, there have been a few attempts to study these evolutionary processes directly in the wild [9-13] or to use maximum likelihood methods to infer the causes contributing to the former effects [14-18].

One of the most appropriate statistics to estimate sexual selection effects is the cross-product estimator (W), which represents the maximum likelihood fitness estimator of one class relative to another [4,7,19]. Sexual isolation estimators try to measure the relative importance of homotypic mating pairs (those between individuals with the same type) in relation to the heterotypic ones (between different types). However, there has been less agreement about the best estimator for sexual isolation effects [reviewed in [4,5,7,20]]. Recently, the statistical properties of all known sexual isolation estimators have been compared [21], revealing that three estimators should be preferentially used: *I*_*PSI*_, *Yule's V *and *YA*.

Complementary pairwise sexual selection and sexual isolation estimators have also been proposed to study mating behaviour in mating frequency data [7]. The *PSI*, *PSS *and *PTI *coefficients, calculated for each combination of mating types in a multiple choice design, represent the sexual isolation, sexual selection and total deviations of each pair combination from the expectations under random mating. In addition, the *PSS *coefficient is an additive decomposition of the cross-product estimator, thus incorporating its advantages [7]. The *PSI *and *PSS *coefficients have been used to distinguish between biological mechanisms acting in nature [12].

The statistical significance for both sexual selection and sexual isolation effects has been assessed with Chi-square or G (likelihood ratio) tests [22,23]. Theoretical resampling variances for some of these statistics have been already described [21]. Additionally, bootstrapping has been also proposed for *I*_*PSI *_and pairwise estimators, giving identical results to those from parametric inference when experimental replication was available [6].

Although one MSDOS program exists that calculates some of the above statistics [6,11], we do not know of any WINDOWS program that includes a comprehensive representation of estimators and statistical tests for the study of sexual selection and sexual isolation. JMATING has been developed to fill this gap.

## Implementation

JMATING is a program written in Java. The java virtual machine (JVM) is needed to run the program, and can be freely downloaded [24]. Once a JVM is properly installed, the program should be able to run in different platforms like Windows, Linux or MacOS X. The user can input mating frequency data manually or from a text file in a specific format [see examples in Additional file 1]. At any time, the data loaded into the program can be saved to a file. There is no limit for the number of species or specimen types but more than 100 mating types will delay significantly the computation time especially for bootstrapping. The data table can be edited by the user and the statistics recomputed with the new data. The results will always appear in an editable panel, which content can be saved to a file. Data can be integer or real numbers, e.g. 1.5 observed matings. There are two reasons for this. First, during the analysis of hermaphrodite data, heterotypic pair counts are typically divided by 2 [25]. Second, the bootstrap test can not check the *PTI *or *PSI *statistics if there are zero observed mating pairs in a particular cell. However, the user can change the bootstrap settings given the program the possibility to change zeros by 0.5 or 1 during bootstrapping, as suggested by Coyne et al. [6]. The default number of bootstrap iterations is 10,000, while the maximum is 100,000.

## Results and discussion

### Available estimators of sexual selection and isolation effects

**Figure 1:**
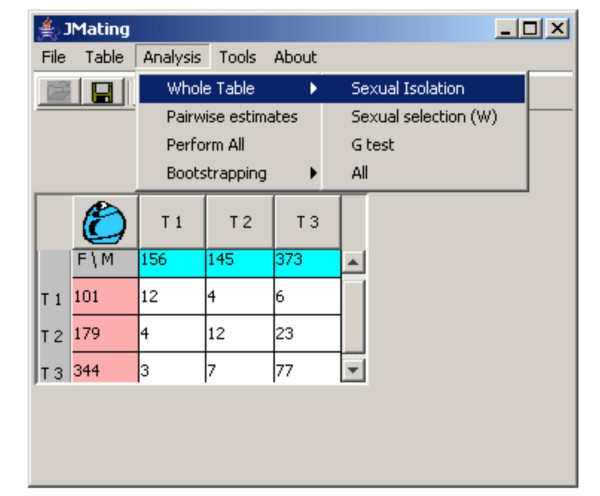
Example 1 of multiple choice data obtained in a field study where the frequency of three morphs and their corresponding mating pairs were recorded [12]. Coloured marginal cells represent the frequency of the three mating types (T1, T2 and T3) in males (blue) or females (pink) sampled. The numbers within the cells in the table represent the observed mating pairs for each particular combination of mating types and sexes.

The analyses implemented in JMATING focus on mating frequency data. The program admits complex tables with up to an indeterminate number of mating types (only limited by the computer memory and speed) and different choice designs: multiple choice, male choice, female choice or no choice designs. We provide two example data sets [Additional file 1]: Example 1 is from a descriptive study made in the wild [12], where two morphs of *Littorina saxatilis *(RB and SU) meet and hybridize on some micro-habitats from the rocky shore (Figure 1); Example 2 was obtained from a multiple choice experiment in the laboratory using two species of *Drosophila *(Table 2a in [6]).

We will use Example 1 to show some features of the analyses available in JMATING (Figure 1). Coloured rows and columns represent the number of specimens of the different types (mating types) used in the experiment. In Example 1 they represent morph frequencies sampled in the wild. For convenience rows always correspond to females (pink) and columns to males (blue). Numbers within the non-coloured cells represent the different mating pairs sampled in the wild (or obtained during the choice experiment, as in Example 2). Two different kinds of analyses are available: global and pairwise estimates.

**Figure 2:**
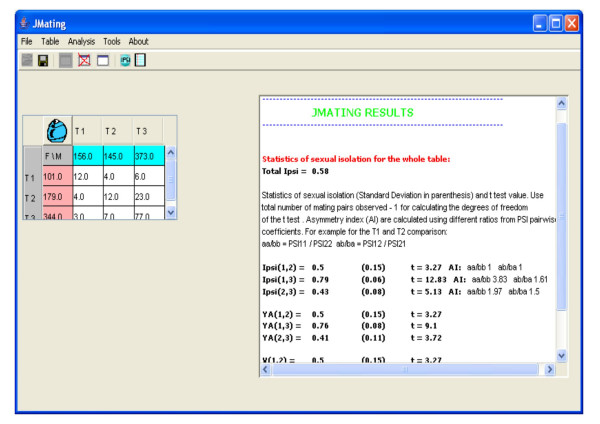
Sexual isolation global analysis for Example 1 (see text).

The global analysis provides the best available estimators of sexual isolation (*I*_*PSI*_, *Yule's V *and *YA*) [7] and sexual selection (W) [4]. The sexual isolation estimators can only be calculated, in principle, for each pair combination of types (in the example: T1 *versus *T2, T1 *versus *T3 and T2 *versus *T3; see Figure 2). The theoretical resampling standard deviation for each estimator is also provided following the formulae given by different authors [21]. The analysis also includes the calculation of the asymmetry of the deviations from random mating in homotypic and heterotypic pairs. For example, for the combination of T1 and T3, this index would be *PSI*_*11*_/*PSI*_*33 *_and *PSI*_*13*_/*PSI*_*31 *_for homotypic and heterotypic pairs, respectively (*PSI *estimates the deviations from random mating for each pair type; see below) [7]. Sexual selection estimates (W) are given for each mate type, in males and females separately, relative to the type with the highest sexual fitness [4] (Figure 2).

Additionally, the program estimates the global sexual isolation using a modification of the *I*_*PSI *_estimator: the deviations from random mating in homotypic (∑(*PSI*_*ii*_)) and heterotypic (∑(*PSI*_*ij*_)) pairs are weighted by the number of mating clases.

Total IPSI=((n−1)×∑(PSIjj))−∑(PSIij)((n−1)×∑(PSIjj))+(∑(PSIij))
 MathType@MTEF@5@5@+=feaafiart1ev1aaatCvAUfKttLearuWrP9MDH5MBPbIqV92AaeXatLxBI9gBaebbnrfifHhDYfgasaacH8akY=wiFfYdH8Gipec8Eeeu0xXdbba9frFj0=OqFfea0dXdd9vqai=hGuQ8kuc9pgc9s8qqaq=dirpe0xb9q8qiLsFr0=vr0=vr0dc8meaabaqaciaacaGaaeqabaqabeGadaaakeaacqqGubavcqqGVbWBcqqG0baDcqqGHbqycqqGSbaBcqqGGaaicqWGjbqsdaWgaaWcbaGaemiuaaLaem4uamLaemysaKeabeaakiabg2da9maalaaabaWaaeWaaeaacqGGOaakcqWGUbGBcqGHsislcqaIXaqmcqGGPaqkcqGHxdaTdaaeabqaamaabmaabaGaemiuaaLaem4uamLaemysaK0aaSbaaSqaaiabdQgaQjabdQgaQbqabaaakiaawIcacaGLPaaaaSqabeqaniabggHiLdaakiaawIcacaGLPaaacqGHsisldaaeabqaamaabmaabaGaemiuaaLaem4uamLaemysaK0aaSbaaSqaaiabdMgaPjabdQgaQbqabaaakiaawIcacaGLPaaaaSqabeqaniabggHiLdaakeaadaqadaqaaiabcIcaOiabd6gaUjabgkHiTiabigdaXiabcMcaPiabgEna0oaaqaeabaWaaeWaaeaacqWGqbaucqWGtbWucqWGjbqsdaWgaaWcbaGaemOAaOMaemOAaOgabeaaaOGaayjkaiaawMcaaaWcbeqab0GaeyyeIuoaaOGaayjkaiaawMcaaiabgUcaRmaabmaabaWaaabqaeaadaqadaqaaiabdcfaqjabdofatjabdMeajnaaBaaaleaacqWGPbqAcqWGQbGAaeqaaaGccaGLOaGaayzkaaaaleqabeqdcqGHris5aaGccaGLOaGaayzkaaaaaaaa@769C@

being *n *the number of different mating types used in the experiment and *PSI*_*jj *_and *PSI*_*ij *_are the sexual isolation estimates for homotypic and heterotypic pair combinations, respectively. To our knowledge, this is the first time that such estimator is proposed and has the advantage to present an overall estimation of sexual isolation when multiple mating types are being used.

**Figure 3:**
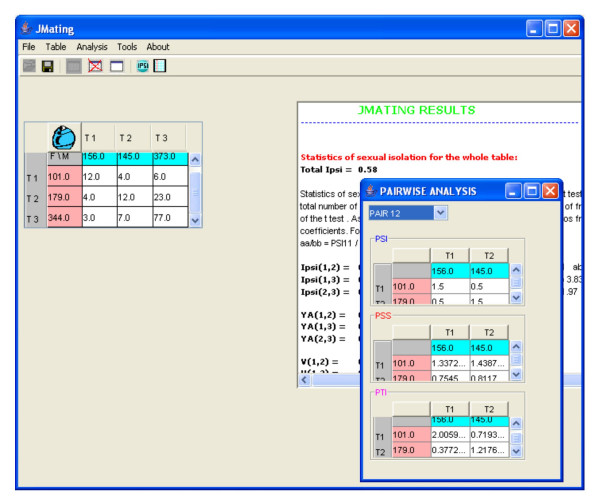
Example of the pairwise analysis for the mating types 1 and 2. It calculates the statistics *PTI*, *PSI *and *PSS *as if only data from mating types 1 (T1) and 2 (T2) were available.

The program also gives the pairwise estimates of total (*PTI*), sexual isolation (*PSI*) and sexual selection (*PSS*) effects from mating frequency data [described in [7]] (Figure 2). The *PSI *coefficients are the sexual isolation effects for each pair. The *PSS *coefficients represent the sexual fitness of each pair, and are an additive decomposition of the cross product estimator (W). The *PTI *coefficients, obtained from the product of *PSI *and *PSS *coefficients, represent the combined sexual selection and sexual isolation effects. All these coefficients can be calculated for the whole data set (all mating types) or comparing exclusively data from a given pair of mating types (Figure 3).

### Available statistical tests

Three different types of statistical tests can be accomplished with JMATING. First, a non-parametric *G *test is available to check for the whole data set if the sexual isolation and sexual selection effects (or both taken together) are significant. The *G *test has additive properties (Sokal and Rohlf, 1996), and thus it can be decomposed additively into the sexual selection (*G*_*S*_) and sexual isolation (*G*_*I*_) components. This decomposition was developed for laboratory experiments, like Example 2, but it can be used for wild data (like Example 1) if the estimates of the morphs in the population are based on large sample sizes (> 30 for each trait and sex). The program gives the value of *G*_*S *_and *G*_*I*_, and their degrees of freedom, as well as their combined effects (*G*_*T *_= *G*_*S *_+ *G*_*I*_), and they can be compared with a χ^2 ^distribution with their corresponding degrees of freedom. Second, JMATING also gives the theoretical sampling distribution for *I*_*PSI*_, *YA *and *V *indexes, allowing the use of a t-test for classical parametric inference [26]. This approach, however, has a high false positive rate [21]. Third, JMATING also provides the bootstrap probability for rejecting the null hypothesis. This alternative is rather conservative [21], and so that we recommend not to use multitest corrections for the bootstrap probability values obtained with our program unless a great number of tests (> 10) are being performed.

JMATING resamples 10,000 times the observed values of mating pairs in order to estimate the bootstrap sampling distribution for the estimator (*I*_*PSI*_, *YA *and *Yule's V*). Then the program calculates the bootstrap average and standard deviation as well as the two-tail probability of getting a sexual isolation estimate significantly different from zero (equivalent to random mating). JMATING can also calculate the bootstrap mean, standard deviation of the cross-product (W) estimators, as well as its (one tail) probability of getting values significantly smaller than 1 (in our case we consider values of W larger than 1 because we use the largest W as the reference value). Notice that the frequency of mating types in the population (in blue and pink cells) are not resampled, because we assume that they are the population frequencies in the experiment. This is only true in laboratory experimental data (Example 2). However, we allow the option to change this and to resample also non-mated data in the case of field experiments (like in Example 1).

Additionally, JMATING also calculates the bootstrap mean, standard deviation and probabilities for *PTI*, *PSI *and *PSS *coefficients. The program resamples 10,000 times the observed frequencies for getting *PTI *coefficients and the observed and the expected frequencies (assuming random mating) from mated data when estimating the *PSI *and *PSS *coefficients. This procedure is somewhat more conservative than resampling exclusively the observed mating pairs [12], but it allows getting independent bootstrapping for *PSI, PSS *and *PTI *coefficients. The latter is convenient if these coefficients are going to be analysed together.

## Conclusion

JMATING implements a complete analysis of sexual selection and sexual isolation effects from laboratory or field mating frequency data. The software permits a battery of complementary tests, including bootstrapping. We believe that it will be helpful for those researchers interested on the evolution of reproductive isolation and the study of sexual selection.

## Availability and requirements

JMATING is freely available from . The software is written in Java, thus reading the java virtual machine (JVM), which can be freely downloaded from . The JMATING software is provided with no guarantee or warranty of any kind. It may be distributed under the terms of the GNU General Public License.

## Authors' contributions

A.C.-R. is the exclusive programmer of JMATING, although the algorithms and methods were implemented in collaboration with E.R.-A, which also wrote the manuscript.

## Supplementary Material

Additional File 1 The program includes one file with two examples of input data for JMating. Example 1: This example represents wild mating data for three ecotypes (mating types) that meet and mate in a particular hybrid zone [12]. Example 2: This example is a laboratory multiple choice experiment between two *Drosophila *species (mating types) that can meet and hybridize in the wild [6]. Click here for file
